# Assessing Visual Fields in Patients with Retinitis Pigmentosa Using a Novel Microperimeter with Eye Tracking: The MP-3

**DOI:** 10.1371/journal.pone.0166666

**Published:** 2016-11-28

**Authors:** Nozomi Igarashi, Masato Matsuura, Yohei Hashimoto, Kazunori Hirasawa, Hiroshi Murata, Tatsuya Inoue, Obata Ryo, Makoto Aihara, Ryo Asaoka

**Affiliations:** 1 Department of Ophthalmology, The University of Tokyo, Tokyo, Japan; 2 Orthoptics and Visual Science, Department of Rehabilitation, School of Allied Health Sciences, Kitasato University, Kanagawa, Japan; University of Florida, UNITED STATES

## Abstract

**Purpose:**

The purpose of the current study is to investigate the test-retest reproducibility of visual fields (VFs) measured with the MP-3 microperimeter, in patients with retinitis pigmentosa (RP).

**Method:**

VFs were twice measured with the MP-3 and also the Humphrey Field Analyzer, using the 10–2 test grid pattern in both perimeters, in 30 eyes (15 right and 15 left eyes) of 18 RP patients (11 males and 7 females). Test-retest reproducibility was assessed using the mean absolute deviation (MAD) measure at all 68 points in the test grid. Reproducibility was also evaluated using the intraclass correlation coefficient (ICC) of VF sensitivities.

**Result:**

The mean sensitivity measured in the HFA 10–2 was significantly higher than that measured in the MP-3 in both the first and second VF tests (p <0.0001, linear mixed model). The MAD was 2.4±0.6 [1.1 to 3.6] dB for MP-3 and 2.4±0.9 [1.1 to 5.1] dB for HFA 10–2, which was not significantly different (p = 0.76, linear mixed model). The ICC value associated with the MP-3 VFs was 0.81±0.13 [0.49 to 0.98], which was significantly larger than that observed for the HFA 10–2 VFs: 0.77±0.19 [0.20 to 0.94] (p = 0.043, linear mixed model).

**Conclusion:**

The MP-3 microperimeter appears to be useful to evaluate central visual function in RP eyes, exhibiting test-retest reproducibility that is equal to, or better than, that observed in HFA 10–2 VFs.

## Introduction

It is estimated that 150 million people are visually disabled in the world. [1] According to the World Health Organization, in developed countries, glaucoma and other chorioretinal or optic nerve diseases are the main causes of visual impairment.[2] Retinitis pigmentosa (RP) is an inherited progressive retinal disease characterized by a loss of photoreceptors, which eventually leads to central visual loss.[3, 4] Diagnosis of the disease is usually made based on the presence of nyctalopia, visual field (VF) constriction, bone spicule pigmentation, and a reduction in electroretinograms. In RP patients, retinal degeneration begins with a loss of the rod photoreceptors associated with nyctalopia. As the disease advances, the cone photoreceptors are also involved, which leads to severe loss of visual function. Damage to the VF usually begins in the periphery and spreads toward the central area; thus visual acuity (VA) tends to be preserved until the latter stages of the disease.

Damage to the central VF of RP patients is usually assessed using a static automated perimeter (SAP), such as the Humphrey Field Analyzer (HFA, Carl Zeiss Meditec AG, Dublin, CA, USA). However, measured VF sensitivity can be largely influenced by measurement noise which hampers detecting disease progression.[5, 6] Recent studies have proposed statistical models that can account for this measurement noise, thereby enabling more robust diagnoses of progression and more accurate predictions of future progression.[7, 8] There are many different causes for the variability associated with the VF measurement. For instance, it is widely acknowledged that patient fatigue is related to unreliable VF measurements and measurement noise. In addition, we have recently reported that eye movements during the VF test are closely related to unreliable VF results and under-estimation of VF sensitivity.[9, 10]

The MP-3 microperimeter (Nidek co.ltd, Aichi, Japan) is a new perimetry instrument. Unlike its predecessor, the MP-1, the MP-3 has a wide dynamic range and retinal sensitivity between 0 and 34 dB can be measured on its background luminance of 31.5 ASB, which is identical to that with the HFA. Another important feature of this microperimeter is that target light is projected onto the retina rather than a screen like in the HFA. The position of the retina is therefore tracked so that target presentations can be automatically aligned, and the exact same location is stimulated at each target presentation. In this manner, we would expect to observe highly reproducible measurements of retinal sensitivity. The purpose of the current study is to investigate test-retest reproducibility in the MP-3 and conventional HFA, in patients with RP.

## Method

The study was approved by the Research Ethics Committee of the Graduate School of Medicine and Faculty of Medicine at The University of Tokyo. Written consent was given by patients for their information to be stored in the hospital database and used for research. This study was performed according to the tenets of the Declaration of Helsinki.

### Subjects

30 eyes (15 right and 15 left eyes) of 18 RP patients (11 males and 7 females), diagnosed based on clinical examination, VF measurements, and electrophysiology were included in the study. All patients were prospectively recruited at the retina clinic in The University of Tokyo Hospital. Each patient underwent VF testing twice with each of the HFA (10–2 Swedish Interactive Threshold Algorithm, SITA, standard program) and MP-3, within a three month period.

All patients enrolled in the study fulfilled the following criteria: (1) RP was the only disease causing VF damage; (2) patients were followed for at least 6 months at The University of Tokyo Hospital and underwent at least two VF measurements prior to this study; (3) measured refraction was between -6 and +3 diopter (D).

### HFA 10–2 measurement

A white-on-white HFA 10–2 measurement was carried out with the Swedish Interactive Threshold Algorithm Standard (SITA) test and the standard Goldmann III stimulus size. Only reliable VFs were used in the analyses, defined as a fixation loss (FL) rate < 20% and a false-positive (FP) rate < 15%, following the criteria used by the HFA software; false negative (FN) rate was not used as an exclusion criterion.

### MP-3 measurement

**Fig 1:**
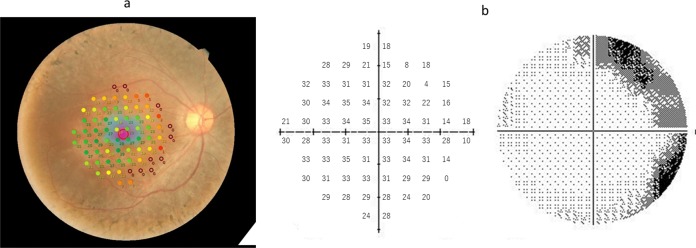
Example VF measurement with the MP-3 and the HFA Perimetry results of a 26 year old female with RP, for the differential light thresholds with a: MP-3 and b: HFA. HFA: Humphrey Field Analyzer, RP: retinitis pigmentosa

All patients had a pupil size that was larger than 4 mm in diameter, which is required for the MP-3 measurement. Similar to the HFA measurement, the MP-3 measurement is based on a 4–2 full threshold staircase strategy using the standard Goldmann III stimulus size. The 68 measured test points in the MP-3 are identical in position to those with the HFA 10–2 test grid (**Fig 1**). The fixation target, a 1° diameter red circle, and the background luminance was set at 31.4 asb. The maximum luminance of the MP-3 is 10,000 asb, and thus the stimulus dynamic range is between 0 and 34 dB. MP-3 examinations were performed in a dim room, similarly to the HFA 10–2 measurement. The order of HFA 10–2 and MP-3 measurements was decided randomly in each patient. Similarly to HFA 10–2, only reliable VFs were used in analyses: a fixation loss (FL) rate < 20% and a false-positive (FP) rate < 15%.

### Statistical analyses

Test-retest reproducibility, of each instrument, was assessed using the mean absolute deviation (MAD) statistic of the 68 threshold values in the two test–retest VFs. Reproducibility was also evaluated using the intraclass correlation coefficient (ICC) of the VF sensitivities of the repeated VF measurement. Then comparisons between HFA 10–2 and MP-3 were made using linear mixed modeling, whereby patients and eyes were treated as random effects.

All analyses were performed using the statistical programming language ‘R’ (R version 3.1.3; The Foundation for Statistical Computing, Vienna, Austria).

## Results

Characteristics of the study subjects are summarized in **Table 1**.

**Table 1:** Patient demographics. SD: standard deviation, HFA: Humphrey Field Analyzer

Age, mean±SD [range]	46.3±17.3 [20 to 78]
Sex, Male:Female	11:7
Eye, Right:Left	15:15
Visual acuity, LogMar, mean±SD [range]	-0.087±0.23 [-0.18 to 1.0]
Refraction, D, mean±SD [range]	-1.6±2.9 [-9.4 to 2.5]
MD (Mean deviation) of 1st HFA 10–2, dB, mean±SD [range]	-12.0±77.5 [-29.7 to 1.6]
MD (Mean deviation) of 2nd HFA 10–2, dB, mean±SD [range]	-11.9±7.8 [-30.9 to 1.2]

The measurement duration with HFA 10–2 was 6 minutes and 54 seconds (6m54s) ± 1m21s [4m17s to 10m13s]. A significantly longer measurement duration was needed for the MP-3 test: 15m54s ± 3m29s [10m51s to 28m6s] (p<0.001, linear mixed model).

**Fig 2:**
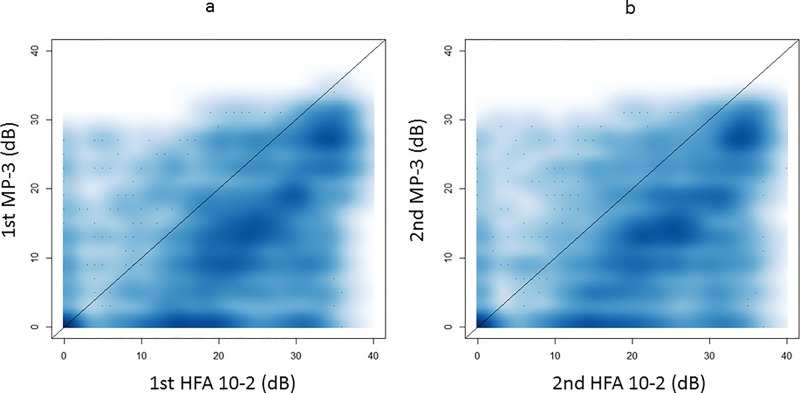
The relationship between the measured sensitivities with MP-3 and HFA 10–2. The relationships between the measured sensitivities with MP-3 and HFA 10–2 are illustrated. a: Comparison in the first measurement and b: comparison in the second measurement. HFA: Humphrey Field Analyzer

**Fig 3:**
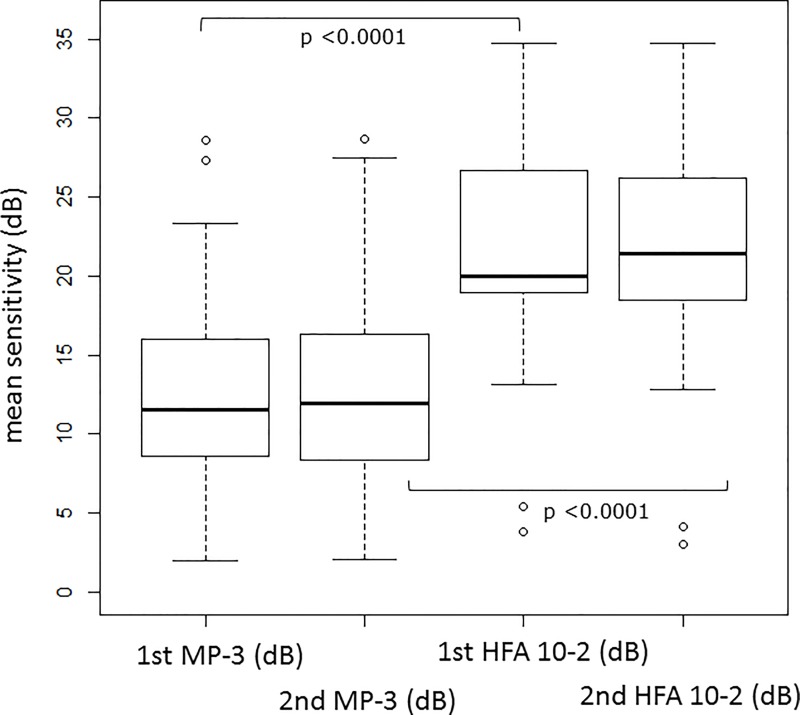
The boxplot of the mean sensitivity with and MP-3 HFA 10–2 The rectangle shows the first and third quartiles and also the median inside. The mean sensitivity with HFA 10–2 was 21.6±7.3 [3.8 to 34.8] (1st measurement) dB and 21.7±7.5 [3.0 to 34.7] dB (2nd measurement), respectively, and 12.7±6.3 [1.9 to 28.5] dB (1st measurement) and 12.7±6.4 [2.1 to 28.7] dB (2nd measurement), respectively, with MP-3. The mean sensitivity was significantly higher with HFA 10–2 compared to MP-3 in both first and second measurements (both p <0.0001, linear mixed model). There was not a significant difference in the mean sensitivity values of the first and second HFA 10–2 (p = 0.57) and also first and second MP-3 (p = 0.75) HFA: Humphrey Field Analyzer

**Fig 2** illustrates the relationship between sensitivities recorded with the HFA and the MP-3, for the first and second measurements. **Table 2** shows the mean sensitivity with HFA 10–2 and MP-3 at the first and second measurements. The mean sensitivity was significantly higher with HFA 10–2 compared to MP-3 in both the first and second measurements (both p <0.0001, linear mixed model). There was not a significant difference in the mean sensitivity values of the first and second HFA 10–2 (p = 0.57) or the first and second MP-3 (p = 0.75): see **Fig 3**.

**Table 2:** Mean sensitivity with each perimeter. SD: standard deviation, HFA: Humphrey Field Analyzer

Perimetry	No	values (mean±SD [range])
HFA 10–2	1st	21.6±7.3 [3.8 to 34.8]
	2nd	21.7±7.5 [3.0 to 34.7]
MP-3	1st	12.7±6.3 [1.9 to 28.5]
	2nd	12.7±6.4 [2.1 to 28.7]

**Fig 4:**
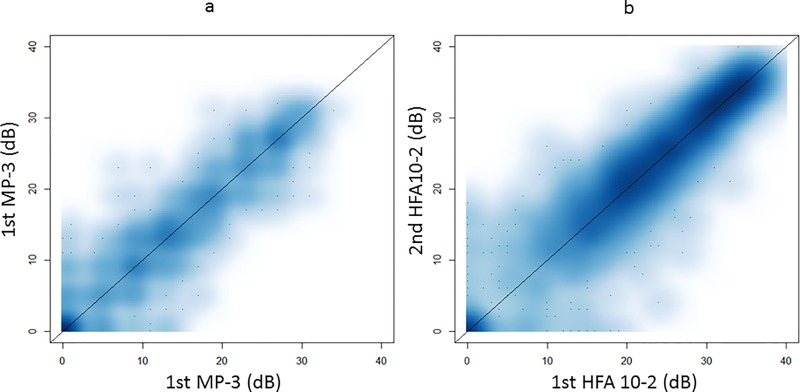
The relationship between the sensitivities in the first and second measurements with MP-3 and HFA 10–2. The MAD (mean absolute difference) was 2.4±0.6 [1.1 to 3.6] dB for MP-3 and 2.4±0.9 [1.1 to 5.1] dB for HFA 10–2, which was not significantly different (p = 0.76, linear mixed model). HFA: Humphrey Field Analyzer

**Fig 5:**
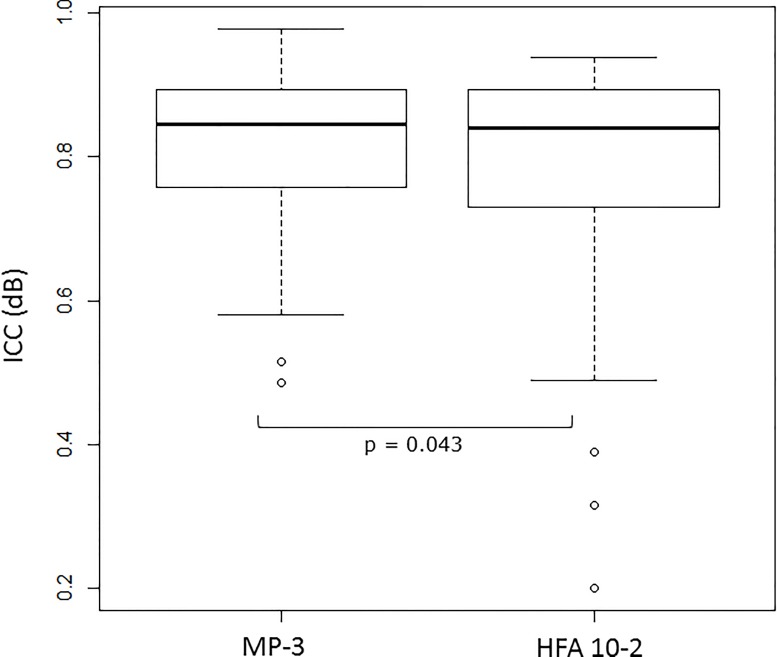
Comparison of ICC values between MP-3 and HFA 10–2 The ICC values associated with the MP-3 between the first and second measurements were 0.81±0.13 [0.49 to 0.98] which was significantly larger than that with HFA 10–2: 0.77±0.19 [0.20 to 0.94] (p = 0.043, linear mixed model). ICC: Intraclass correlation coefficient, HFA: Humphrey Field Analyzer

**Fig 4** illustrates the relationship between the sensitivities recorded in the first and second measurements with each perimeter. The mean absolute variability was 2.4±0.6 [1.1 to 3.6] dB for MP-3 and 2.4±0.9 [1.1 to 5.1] dB for HFA 10–2, which was not significantly different (p = 0.76, linear mixed model). The ICC value associated with the MP-3 measurements was 0.81±0.13 [0.49 to 0.98], which was significantly larger than that associated with the HFA 10–2 tests: 0.77±0.19 [0.20 to 0.94] (p = 0.043, linear mixed model); see **Fig 5**.

## Discussion

In the current study, test-retest VF measurements were carried out using HFA and MP-3 perimeters, in patients with RP. Test sensitivities were significantly lower in MP-3 than in HFA. Test-retest reproducibility was assessed by calculating the MAD and also the ICC statistic. Both perimeters exhibited relatively small absolute differences, however, the ICC was significantly improved with the MP-3 perimeter compared to the HFA.

Perimetry measurements are inherently associated with measurement noise, due to a number of different reasons, including short-[11] and long-term fluctuation,[12] loss of patients’ concentration and eye movements.[9, 10] The MP-3 microperimeter predefines retinal positions and is equipped with an auto-tracking system. As a result, a stimulus can be projected onto these predefined retinal positions mitigating the problem of eye movements during the VF measurement. Improving test-retest reproducibility is crucial for accurately and quickly detecting VF progression.[5] A limitation of the current MP-3 model is that only a full threshold strategy is available, which considerably increases measurement duration–compared with the HFA SITA strategy–and can affect test-retest reproducibility.[13] Nonetheless, in the current study, MAD was not significantly different between HFA 10–2 and MP-3 tests, and, moreover, the ICC was significantly better with the MP-3 test than it was with the HFA 10–2 test. Further improvements in MP-3 test-retest reproducibility should be possible if a quicker measurement strategy was employed. It should also be noted that the MP-3 perimeter’s auto-tracking system requires considerable time to align the eye’s fixation position, adding to patient fatigue. It may be possible to shorten this time by loosening the auto-tracking criteria to determine fixation position. However, it will be important to determine how any change in the criteria affects test reproducibility, striking the right balance between reproducibility of the stimulus position on retina and shortening test duration. Also a newer software is planned to be implemented in the MP-3 (personal communication with Nidek Co.ltd) which has shorter duration between each target presentation. The efficacy of this new software should be investigated in the future.

In the current study, VF sensitivities from MP-3 were dramatically and significantly lower than HFA 10–2 (**Fig 2** and **Table 2**), despite an identical background luminance. The dynamic range of the MP-3 is slightly smaller than that in the HFA, which may explain some of the difference in sensitivities; this is particularly important at locations near to fixation where retinal sensitivity higher than 34 dB is more likely to be observed. However, as shown in **Fig 2**, MP-3 sensitivity tends to be smaller than HFA 10–2 sensitivity in the range 10 to 30 dB. One possible reason for reduced sensitivity in the MP-3 is its prolonged measurement duration, because it has been reported that this is associated with lower recorded VF sensitivity.[14, 15] However, this difference in duration is unlikely to explain all of the reduction in sensitivity, which was equal to approximately 9 dB in the current study. Further, in a previous study, sensitivity difference between full threshold and SITA strategies was merely 3 dB.[13] In perimeters without auto-tracking, such as the HFA, small (less than 3°) eye movements during the VF measurement cannot be avoided, even in well-trained healthy subjects.[16, 17] Thus, the Goldmann size III stimulus (with a diameter of 0.43 degrees) is projected onto the retina anywhere within at least 3 degrees of the designed location. This phenomenon is not observed with the MP-3, because its stimulus is projected onto a particular position of the retina, adjusted for eye movements according to perimeter’s auto-tracking function. This suggests that retinal sensitivity is accurately measured at an exact point with the MP-3, but this may not be the case with the HFA. Furthermore, since the exact same point is stimulated in the MP-3, this may lead to light adaptation at the test point causing retinal sensitivity to become lower at that point due to repeated target presentations. This would be exaggerated with RP patients, compared to glaucoma patients, because the photoreceptor cell itself is the site of disease in RP, whereas it is the retinal ganglion cell in glaucoma.

Retinal sensitivity is also decreased at the site of a retinal vessel (angioscotoma).[18, 19] In the MP-3 measurement, the stimulus at some of the test points may be repeatedly projected onto a retinal vessel. Thus, the reproducible–but lower VF sensitivity–measured with MP-3 may be because it truly reflects retinal damage more sensitively than HFA 10–2, but also because it is affected by other aspects, such as an angioscotoma. A future study should investigate the structure-function relationship in the MP-3 and the HFA 10–2, measuring retinal thickness using optical coherence tomography (OCT); in particular the thickness between the retinal pigment epithelium and the outer plexiform layer.[20] In a recent study by Hirooka et al., the measured sensitivities were compared between HFA 10–2 and MP-3 in patients with glaucoma.[21] Similarly to our result, MP-3 sensitivity was lower than that with HFA 10–2. However, the difference was much smaller than that in the current study (between 4 and 5 dB). The reason for this difference is not entirely clear, but could be associated with the much shorter measurement duration (846.9 seconds on average) in the previous study compared to the current study. Despite the difference in measured sensitivity between MP-3 and HFA 10–2, the structure-function relationship was almost the same for these perimeters. Different results could be observed in eyes with RP, because significant axonal loss precedes the development of visual field defects in glaucoma: as much as 20% of the normal number of cells were gone in locations with a 5 dB sensitivity loss, and a 40% cell loss corresponded to a 10 dB decrease.[22]

Thus, it is clearly of interest to measure the structure-function measurement, however, this study did not include a structural measurement, such as OCT, which is a limitation. A further limitation of the current study was that test points outside the HFA 10–2 grid were not evaluated (the current MP-3 has the maximum field of view of within 20 degrees from central point). In the current MP-3, it is not possible to carry out a VF measurement at all points in the HFA 24–2 grid. A comparison between MP-3 and HFA, in the 24–2 test grid, should be carried out in future if the MP-3 obtains a wider measurement area. Finally, to the best of our knowledge, no study has been carried out to compare the test-retest reproducibility of visual fields in the central 10 degrees between RP and other diseases, such as glaucoma, which should be performed in future, because it is closely related to the ability to detect progression.[5, 6] In addition, RP is a very heterogeneous disease, and hence investigation should be carried out specifying the genetic description of the patients in future.

In conclusion, the good test-retest reproducibility of the MP-3 suggests that it is a useful perimetry to evaluate central visual function in RP eyes. Further studies should be carried out, however, to determine why sensitivities recorded with MP-3 are significantly lower than those observed with HFA. In addition, MP-3 was associated with longer test duration, hence further efforts should be made to overcome this problem

## Supporting Information

S1 File Data analyzed (CSV)
